# Compartmentalization of casein kinase 1 γ CSNK1G controls the intracellular trafficking of ceramide

**DOI:** 10.1016/j.isci.2022.104624

**Published:** 2022-06-16

**Authors:** Asako Goto, Shota Sakai, Aya Mizuike, Toshiyuki Yamaji, Kentaro Hanada

**Affiliations:** 1 Department of Biochemistry and Cell Biology, National Institute of Infectious Diseases, Shinjuku-ku, Tokyo 162-8640, Japan

**Keywords:** Biological sciences, Cell biology, Functional aspects of cell biology

## Abstract

Casein kinase 1 γ (CK1G) is involved in the regulation of various cellular functions. For instance, the ceramide transport protein (CERT), which delivers ceramide to the Golgi apparatus for the synthesis of sphingomyelin (SM), is inactivated when it receives multiple phosphorylation by CK1G. Using human genome-wide gene disruption screening with an SM-binding cytolysin, we found that loss of the *C*-terminal region of CK1G3 rendered the kinase hyperactive in cells. Deletion of the *C*-terminal 20 amino acids or mutation of cysteine residues expected to be palmitoylated sites redistributed CK1G3 from cytoplasmic punctate compartments to the nucleocytoplasm. Wild-type CK1G3 exhibited a similar redistribution in the presence of 2-bromopalmitate, a protein palmitoylation inhibitor. Expression of *C*-terminal mutated CK1G1/2/3 similarly induced the multiple phosphorylation of the CERT SRM, thereby down-regulating *de novo* SM synthesis. These findings revealed that CK1Gs are regulated by a compartmentalization-based mechanism to access substrates present in specific intracellular organelles.

## Highlights

• *C*-terminal region of CSNK1Gs restricts their localization to punctate compartments
• Loss of the kinase compartmentalization causes hyperphosphorylation of CERT
• Compartmentalization of CSNK1G controls ceramide transport and *de novo* SM synthesis

## Graphical abstract

**Figure:**
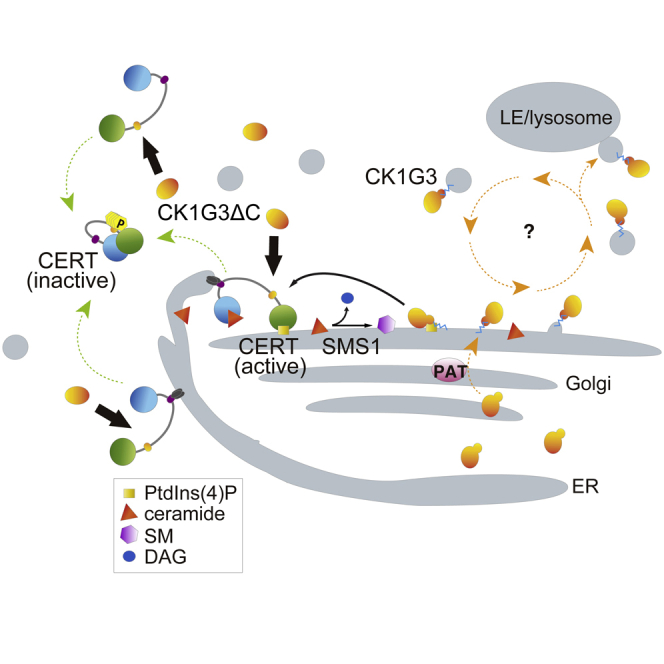

## Introduction

**Figure 1:**
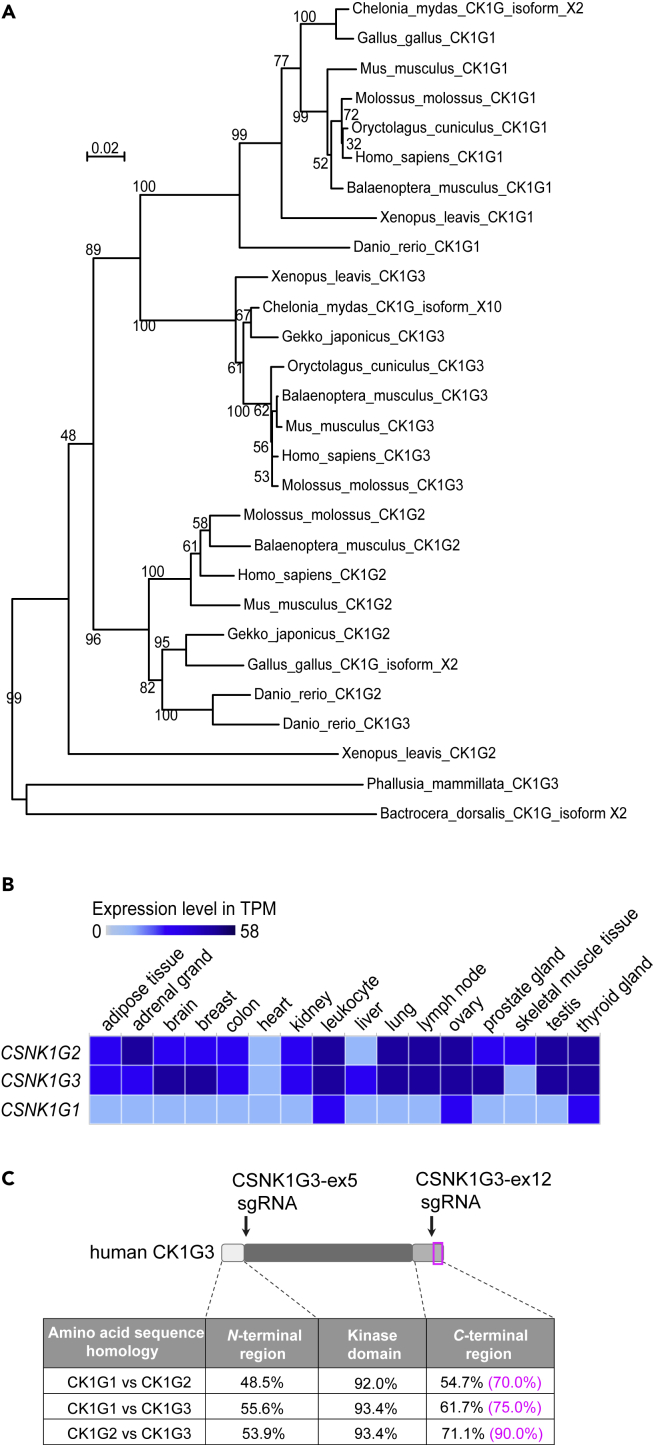
Homology and expression of the casein kinase 1 γ family (A) Phylogenetic analysis of the vertebrate casein kinase 1 γ family proteins. SeaView’s BioNJ algorithm (http://doua.prabi.fr/software/seaview) (Gouy et al., 2010) was used to construct the unrooted tree from evolutionary distance data (Gascuel, 1997). In this analysis, CK1Gs of the invertebrate *Phallusia mammillata* (ascidiacea) and *Bactrocera dorsalis* (fly) were used as out-group sequences. Despite the high homology within their kinase domains, CK1G1, CK1G2, and CK1G3 homologs from various vertebrate organisms were clustered in distinct branches. Accession numbers of proteins used for the phylogenetic analysis are listed in Table S1. (B) Expression levels in transcripts per million (TPM) of human *CSNK1G1*, *CSNK1G2*, and *CSNK1G3* in various tissues. Light and dark colors represent low and high levels of expression, respectively. (C) Amino acid sequence homologies of human CK1G1, CK1G2, and CK1G3. The *C*-terminal 20 amino acid region is indicated with a magenta-colored box. Positions of sgRNAs CSNK1G3-ex 5 and CSNK1G3-ex 12 are indicated with black arrows.

Casein kinase 1 γ (CK1G) is an isoform of the casein kinase 1 (CK1)-serine/threonine protein kinase family, which is widely conserved among animals (Figure 1A). Proteins with conserved motifs for CK1 exist in all cell types in humans, and CK1G regulates various cellular functions (Knippschild et al., 2014). The human genome encodes three different CK1G structural genes, *CSNK1G1*, *CSNK1G2*, and *CSNK1G3,* which are ubiquitously expressed in various tissues at varying levels (Figure 1B, AceView, https://www.ncbi.nlm.nih.gov/IEB/Research/Acembly/; Expression Atlas, https://www.ebi.ac.uk/gxa/home). Their respective protein products CK1G1, CK1G2, and CK1G3 share a highly conserved kinase domain but considerable heterogeneity exists in their *N*- and *C*-terminal regions (Figure 1C), which are supposed to determine the functional specificities of each subtype (Han et al., 2014). Nevertheless, the amino acid sequences of the most distal *C*-terminal region are highly conserved among the three CK1Gs (Figures 1C), although the biological meaning of the conserved *C*-terminal region remains poorly understood.

*De novo* synthesis of sphingomyelin (SM) requires the delivery of ceramide from the ER to the *trans*-Golgi regions, where ceramide is converted to SM. The inter-organelle trafficking of ceramide is mediated by the ceramide transport protein (CERT) (Hanada et al., 2003). When a serine-repeat motif (SRM) of CERT receives multiple phosphorylation (hereafter referred to as hyperphosphorylation), the function of CERT is inactivated (Kumagai et al., 2007). Our previous study to seek factors involved in the synthesis of SM in mammalian cells by screening with the SM-binding cytolysin lysenin revealed that overproduction of CK1G2 resulted in the hyperphosphorylation of the CERT SRM, thereby inhibiting the *de novo* synthesis of SM (Tomishige et al., 2009). Knock-down experiments have suggested that CK1G1 and/or CK1G3 are partially responsible for the hyperphosphorylation of the CERT SRM, whereas CK1G2 likely plays a major role in Chinese hamster ovary-derived CHO cells (Tomishige et al., 2009).

In the current study, after a human genome-wide gene disruption screening approach, we revealed that the *C*-terminal region of the CK1Gs is a determinant of their cytoplasmic punctate compartmentalization and that loss of their *C*-terminus redistributes the kinase to the nucleocytoplasm, which induces the inactivation of CERT. These results suggested that the *C*-terminal region-dependent endomembrane retention of CK1Gs is crucial for the regulatory phosphorylation of specific substrates.

## Results

### *C*-terminal truncation of CK1G3 down-regulates the synthesis of sphingomyelin and confers lysenin resistance

Our recent genome-wide CRISPR/Cas9-mediated gene disruption screening to seek factors involved in lysenin sensitivity suggested CK1G3 as a candidate (Mizuike et al., in preparation). However, this was seemingly contradicted with our previous study indicating that the overproduction of CK1G2 endowed lysenin-resistance to cells (Tomishige et al., 2009). In order to obtain a clue to solve the mechanism by which the *CSNK1G3* gene mutations cause lysenin resistance, we made a list of sgRNA sequences targeting the three human CK1G subtypes, CK1G1, CK1G2, and CK1G3, from the two sgRNA libraries used in our screening experiment. Then, we found that only two out of six sgRNAs targeting *CSNK1G3* were enriched in the screening and that both of the two positive sgRNAs targeted exon 12 of *CSNK1G3*, which encodes the *C*-terminal region of CK1G3 (Table S2). By contrast, none of the sgRNAs that target the kinase domain-encoding exons of *CSNK1G1*, *CSNK1G2,* and *CSNK1G3* were enriched in the screening (Table S2).

**Figure 2:**
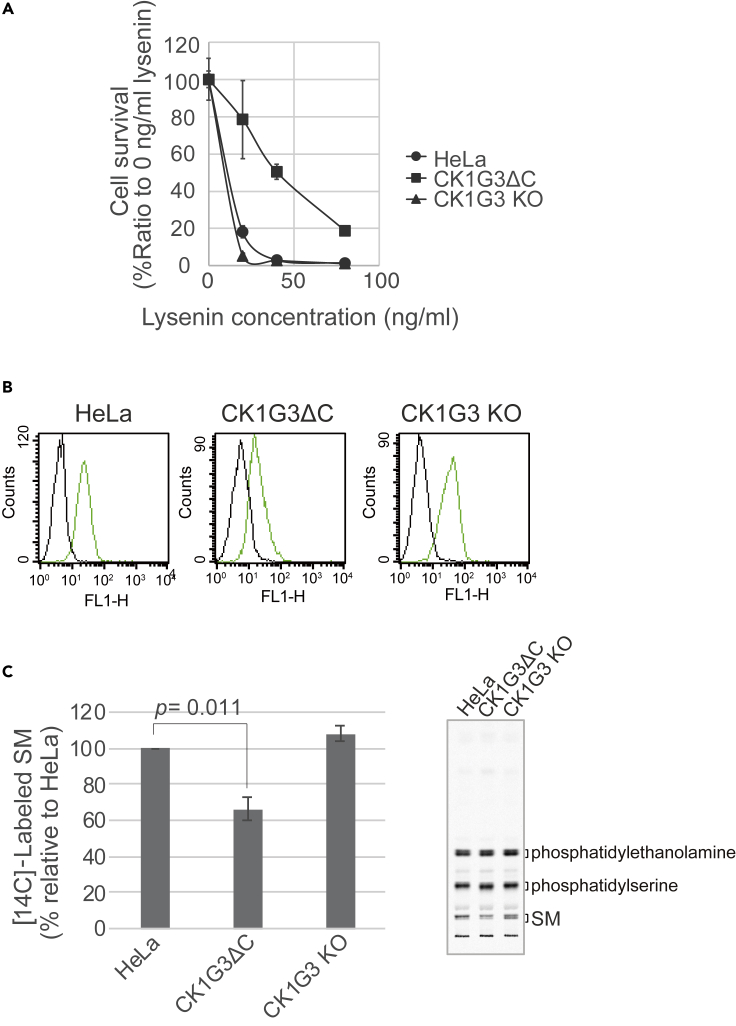
*C*-terminal truncation of the endogenous CK1G3 down-regulates SM synthesis in HeLa cells (A) Lysenin sensitivity assay. Cells were seeded in 12-well plates, cultured overnight, and treated with 0, 20, 40, or 80 ng/mL lysenin for 2 h at 37°C. The cell survival ratio was measured by MTT assay. Results shown are the mean and SEM of three experiments. (B) Trypsinized cells were incubated with GFP-lysenin on ice for 1 h, and the level of GFP-lysenin bound to cells was determined by FACS analysis. Black, without GFP-lysenin; green, GFP-lysenin added. (C) *De novo* synthesis of SM was measured by metabolic labeling of lipids with [^14^C]serine for 24 h. Results shown are the mean and SEM of three experiments. Significance was determined by the two-tailed Student’s *t* test; p values < 0.05 were considered statistically significant.

To verify that lysenin resistance was acquired by the loss of the *C*-terminus but not loss of the kinase domain of CK1G3, we generated two *CSNK1G3* mutant HeLa cell lines using a targeted genome-editing with two sets of sgRNAs. The sgRNA targeting exon 12 of *CSNK1G3* generated a mutant cell line (named CK1G3ΔC) that had short base insertions causing frameshifts in the *C*-terminal regions in two alleles of *CSNK1G3* (Figures 1C and S1). Thus, CK1G3 of CK1G3ΔC cells lacked the *C*-terminal region but retained the intact kinase domain. By contrast, the sgRNA targeting exon five generated a loss-of-function mutant cell line (named CK1G3 KO) that had short deletions in the exon, which caused frameshifts in the kinase domain of CK1G3 (Figures 1C and S1). CK1G3 KO cells were sensitive to lysenin, similar to the parental HeLa cells, whereas CK1G3ΔC cells exhibited a clear resistance (Figure 2A), in line with the results of the genome-wide mutation screening.

Lysenin exerts its cell toxicity through binding to nano-scale clusters of SM at the plasma membrane (Bokori-Brown et al., 2016; Yamaji et al., 1998). We examined the binding of a GFP-fused lysenin to the cell surface of live cells by FACS analysis and found that CK1G3ΔC cells but not CK1G3 KO cells exhibited less lysenin-binding, compared with the parental HeLa cell control (Figure 2B). This result eliminated the possibility that CK1G3ΔC cells were lysenin-resistant despite a normal lysenin-binding ability. Next, we determined whether the synthesis of SM was compromised in CK1G3ΔC cells by the metabolic labeling of lipids with radioactive serine. The *de novo* synthesis of SM was significantly decreased in CK1G3ΔC cells, compared to the parental cells (Figure 2C). By contrast, CK1G3 KO cells had the tendency for a slight increase in SM synthesis (Figure 2C). The synthesis of ceramide and glycerolipids (*i.e.*, phosphatidylserine and phosphatidylethanolamine) was not remarkably affected in CK1G3ΔC or CK1G3 KO cells (Figure 2C), eliminating the possibility that these CK1G3 mutations had non-specific impacts on lipid metabolism. Of note, the synthesis of glucosylceramide in CK1G3ΔC cells was increased, compared with that in the parental control cells and CK1G3 KO cells (Figure S2) probably because repression of the synthesis of SM redirected the common precursor ceramide to the synthesis of glucosylceramide. These results indicate that the CK1G3ΔC mutation resulted in the repression of the *de novo* synthesis of SM, thereby rendering the mutant cells resistant to lysenin.

Mammalian cells have at least two pathways for transporting ceramide from the ER to the Golgi site for SM synthesis: One is the CERT-dependent major pathway and the other is the CERT-independent minor pathway(s), although the identity of the latter pathway remains elusive. Hence, destroying the CERT-dependent pathway (by gene mutations in *CERT1* and with selective inhibitors of CERT) only results in partial repression of the *de novo* synthesis of SM (Hanada et al., 2003; Murakami et al., 2020; Nakao et al., 2019; Yamaji and Hanada, 2014). This resembles the current finding that the *C*-terminal truncation of CK1G3 significantly, but not completely, repressed the synthesis of SM in cells (Figure 2C).

### Ceramide transport protein is hyperphosphorylated in CK1G3ΔC cells

**Figure 3:**
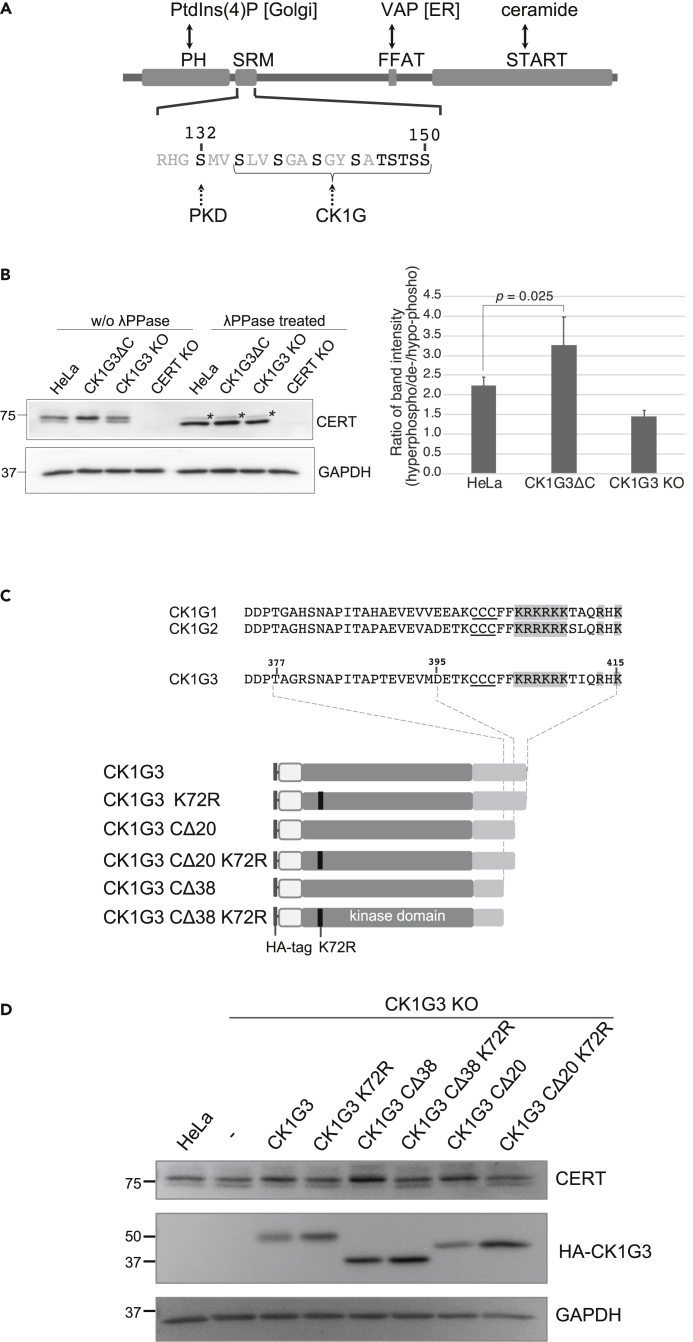
CERT is hyperphosphorylated at the SRM in cells expressing CK1G3ΔC (A) Domains and motifs in CERT. PH, pleckstrin homology domain; SRM, serine repeat motif; FFAT, two phenylalanines in an acidic tract motif; START, steroidogenic acute regulatory-related lipid transfer domain. (B) Phosphorylation of CERT at the SRM in HeLa parental, CK1G3ΔC, and CK1G3 KO cells. Total cell lysates (10 μg protein) with or without λPPase treatment were resolved by SDS-7.5% PAGE and immunoblotted with an anti-CERT antibody or anti-GAPDH. The bands marked with asterisks (∗) represent the dephosphorylated form of the CERT_L_ variant (Murakami et al., 2020). Signal intensity of the bands corresponding to hyperphosphorylated and de-/hypo-phosphorylated forms of CERT was quantified and the ratios were shown in the bar graph. Results shown are the mean and SEM of six experiments. Significance was determined by the two-tailed Student’s *t* test; p values < 0.05 were considered statistically significant. (C) Construction of *C*-terminus deletion and/or kinase-dead K72R mutants of CK1G3. *C*-terminal sequences of the human CK1G1, CK1G2, and CK1G3 are aligned. Putative palmitoylation sites are underlined. Basic amino acids in the *C*-terminus are shaded in gray. (D) Phosphorylation of CERT at the SRM in CK1G3 KO cells stably expressing various CK1G3 constructs with an *N*-terminal HA epitope tag. Lysate fractions prepared from the cells were resolved by SDS-7.5% PAGE and immunoblotted with anti-CERT antibody or anti-GAPDH.

The *N*-terminal pleckstrin homology (PH) domain of CERT preferentially binds to phosphatidylinositol 4-monophosphate [PtdIns(4)P] among various phosphoinositides and serves as a functional module for targeting the *trans*-Golgi regions (Figure 3A) (Hanada et al., 2003). The hyperphosphorylation of the CERT SRM compromises the activity of the CERT PH domain and represses the Golgi-association of CERT (Figure 3A) (Kumagai et al., 2007; Sugiki et al., 2018). When CK1G2 is overproduced, CERT largely shifts to the SRM hyperphosphorylated form, thereby repressing the *de novo* SM synthesis (Tomishige et al., 2009). We examined whether the CK1G3ΔC mutation induced the hyperphosphorylation of CERT by Western blotting analysis (Figure 3B): in the parental HeLa cells, both dephosphorylated and hypo-phosphorylated (de-/hypo-phosphorylated) forms of CERT were observed, but the hyperphosphorylated form was more abundant. The band corresponding to the hyperphosphorylated form of CERT downshifted to the dephosphorylated band when the sample cell lysates were pre-treated with λ protein phosphatase (λPPase) (Figure 3B), verifying the assignments of the bands to the hyperphosphorylated form. By contrast, the de-/hypo-phosphorylated forms were absent or far less detected in CK1G3ΔC cells but tended to slightly increase in CK1G3 KO cells, compared with the parental cell control (Figure 3B). These results revealed that CK1G3ΔC mutation in the human genome induced the hyperphosphorylation of CERT and down-regulated its function to produce SM.

To determine which regions in CK1G3 are crucial for the *C*-terminal truncation-induced gain-of-function, we generated cDNA constructs encoding various CK1G3 mutants with an *N*-terminal HA-epitope, cloned them into a retroviral vector, and stably expressed them in CK1G3 KO cells (Figure 3C). The CK1G3 mutants included two types of *C*-terminal deletion mutants. One was CK1G3 CΔ38, in which the 38 amino acids of the *C*-terminus of the wild-type CK1G3 were truncated by inserting a stop codon; this mutant was a mimic of the genome-edited CK1G3ΔC mutant. We also made CK1G3 CΔ20 with a 20-amino acid-truncation of the *C*-terminus, inspired by a previous study showing that a 20-amino acid-truncation of the *C*-terminus of CK1G1 in *Xenopus* disturbed its substrate phosphorylation (Davidson et al., 2005). Kinase-dead mutants were also constructed by replacing the conserved lysine 72 with arginine (K72R) (Davidson et al., 2005). When ectopically expressed in CK1G3 KO cells, wild-type CK1G3 induced a shift from the de-/hypo-phosphorylated forms to the hyperphosphorylated form of CERT, and this shift was canceled by the kinase-inactivating point mutation, namely CK1G3 K72R (Figure 3D). CK1G3 CΔ38 and CK1G3 CΔ20 induced similar but more prominent shifts, and this effect was also canceled by the K72R mutation. These results indicated that the kinase activity of CK1G3 was indispensable for the *C*-terminal-deleted CK1G3 mutants to induce the hyperphosphorylation of CERT. Because CΔ38 and CΔ20 exhibited similar impacts on the CERT SRM phosphorylation, we focused on CK1G3 CΔ20 for further analysis.

### Altered subcellular localization of CK1G3 owing to the *C*-terminal deletion disables ceramide transport protein to localize at the golgi apparatus

**Figure 4:**
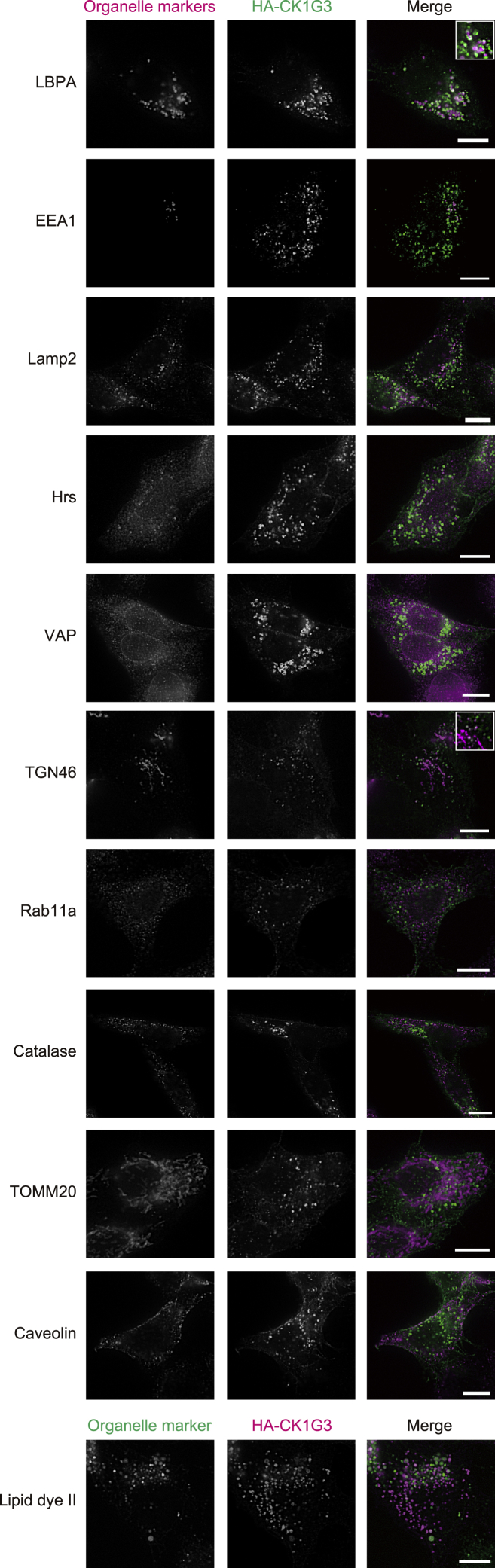
CK1G3 localizes to cytoplasmic punctate compartments Intracellular localization of HA-CK1G3. CK1G3 KO cells expressing HA-CK1G3 were immunostained with HA antibody and antibodies against various organelle markers: LBPA (late endosomes), EEA1 (early endosomes), Lamp2 (lysosomes), Hrs (exosomes), VAP (ER), TGN46 (TGN), Rab11a (recycling endosomes and Rab11a-positive secretory vesicles), catalase (peroxisomes), TOMM20 (mitochondria), caveolin (caveolae), and lipid dye II (lipid droplet). For the visualization of lipid droplets, cells were pre-incubated in a medium containing 100 μM oleic acid for 24 h before fixation. The scale bars indicated in microscopy images represent 10 μm.

**Figure 5:**
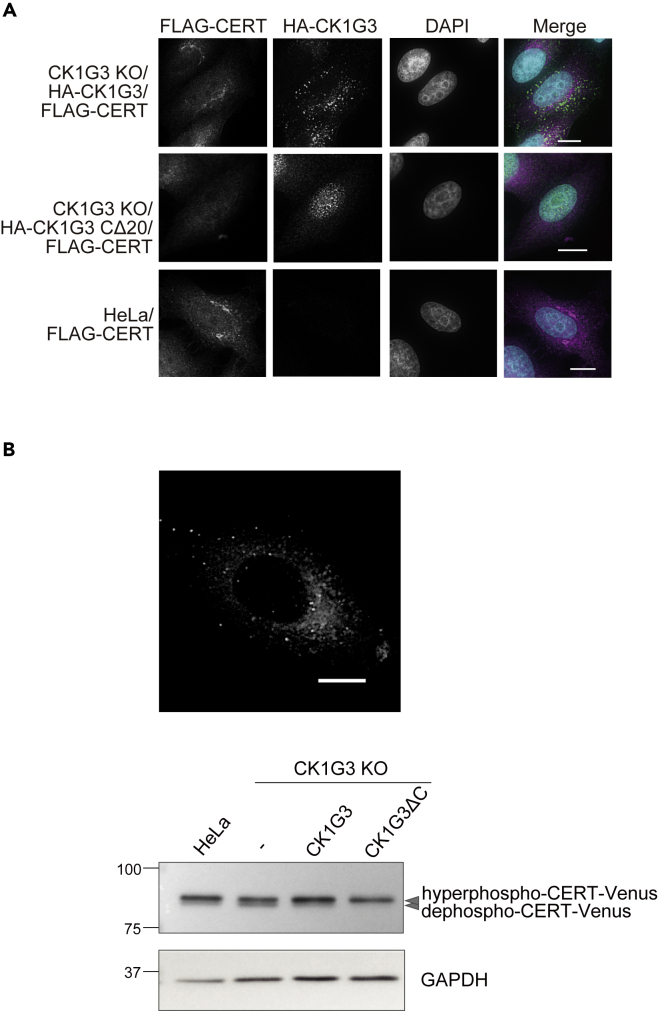
The *C*-terminus-deleted CK1G3 disperses throughout the nucleocytoplasm and highly phosphorylates CERT (A) Effects of *C*-terminal deletion of CK1G3 on the subcellular distributions of CK1G3 and CERT. FLAG-CERT and HA-CK1G3 were immunostained with anti-CERT and anti-HA antibodies, respectively. *Upper panels,* HeLa CK1G3 KO cells expressing HA-CK1G3 wild-type and FLAG-CERT; *middle panels*, HeLa CK1G3 KO cells expressing HA-CK1G3 CΔ20 and FLAG-CERT; *lower panels*, HeLa wild-type cells expressing FLAG-CERT. (B) *Upper panel*: fluorescence signals of CERT-mVenus in HeLa cells. *Lower panel*: hyperphosphorylation of the cytosolic CERT-mVenus construct by CK1G3 CΔ20. Lysate fractions prepared from the indicated cells were resolved by SDS-6% PAGE and immunoblotted with anti-GFP antibody or anti-GAPDH. The scale bars indicated in microscopy images represent 10 μm.

To examine whether the *C*-terminal deletion of CK1G3 affected its subcellular localization, we immunostained CK1G3 and CK1G3 CΔ20 with an anti-HA antibody. As markers for various organelles, we employed lysobisphosphatidic acid (LBPA) (also known as bis(monoacylglycerol)phosphate) for late endosomes, EEA1 for early endosomes, Lamp2 for lysosomes, the hepatocyte growth factor-regulated tyrosine kinase substrate (Hrs) for exosomes, vesicle-associated membrane protein-associated protein (VAP) for the ER, TGN46 for the trans-Golgi network (TGN), Rab11a for recycling endosomes and Rab11a-positive secretory vesicles, catalase for peroxisomes, TOMM20 for mitochondria, caveolin for the plasmalemmal vesicles termed caveolae and lipid dye II for lipid droplets. CK1G3 did not exhibit perfect colocalization with any of the organelle markers tested (Figure 4). CK1G3 was not distributed to the cell surface (Figure 4). The signals mostly appeared as punctate structures, which most frequently overlapped with the late-endosome marker LBPA and less frequently with Lamp2 and TGN46 (Figure 4). The CK1G3-positive punctate structures tended to be located in the vicinity of the exosome marker Hrs (Figure 4), raising the possibility that the CK1G3-positive punctate structures partially included heterogeneous functional zones in the “sorting endosomes,” which were reviewed previously (Naslavsky and Caplan, 2018; Redpath et al., 2020). Overall, these results suggested that CK1G3 was largely distributed to post-Golgi compartments (e.g., late endosomes, recycling endosomes, and lysosomes) as well as less frequently to the distal Golgi compartments (e.g., TGN and *trans*-Golgi cisterna). In contrast, CK1G3 CΔ20 localized to the cytosol and the nucleus, but not to punctate compartments (Figure 5A), indicating that the *C*-terminal region of CK1G3 is a determinant of its subcellular localization.

To investigate whether the altered localization of CK1G3 CΔ20 affected the subcellular localization of CERT, we performed co-immunostaining of CK1G3 or CK1G3 CΔ20 and CERT. Owing to its low expression levels, identifying specific immunofluorescent signals of endogenous CERT was infeasible with the antibodies currently available. Therefore, *N*-terminal FLAG-tagged CERT was overexpressed in CK1G3 or CK1G3 CΔ20 cells. In the parental HeLa cells, FLAG-tagged CERT was distributed in the Golgi apparatus and the cytosol (Figure 5A) (Hanada et al., 2003). The distribution pattern of FLAG-tagged CERT in CK1G3 KO/HA-CK1G3 cells resembled the pattern in wild-type HeLa cells, but its localization to the Golgi was slightly weaker (Figure 5A). As HA-CK1G3 and TGN46 were occasionally colocalized at the perinuclear Golgi region (Figure 4), no clear colocalization of FLAG-CERT with HA-CK1G3 was observed (Figure 5A). This discrepancy will be discussed later in discussion. In CK1G3 KO/HA-CK1G3 CΔ20 cells, CERT was extensively cytosolic and did not localize to the Golgi (Figure 5A).

Because CK1G3 CΔ20 and CERT were distributed to both the cytosol and nucleus (Figure 5A), we attempted to determine whether the CK1G3-dependent hyperphosphorylation of CERT mainly occurs in the cytosol, in the nucleus, or both. For this aim, we employed a *C*-terminal EGFP-tagged CERT that is excluded from the nucleus probably owing to size-limitation in spontaneous transport through nuclear pores (Hanada et al., 2003). We expressed a *C*-terminal mVenus-tagged (an EGFP variant) CERT in HeLa wild-type (Figure 5B upper panel), CK1G3 KO, CK1G3 KO/HA-CK1G3, and CK1G3 KO/HA-CK1G3 CΔ20 cells and examined the phosphorylation status of CERT-mVenus. Comparable to endogenous CERT (Figure 3D), expression of CK1G3 CΔ20 induced a shift from the de-/hypo-phosphorylated form of CERT-mVenus to the hyperphosphorylated form (Figure 5B lower panel). Although the significance of the nucleus localization of CK1G3 CΔ20 was unclear, we concluded that the cytosolic CK1G3 CΔ20 hyperphosphorylated and inactivated the CERT.

### *C*-terminal palmitoylation may be crucial for the cytoplasmic punctate distribution of CK1G3

**Figure 6:**
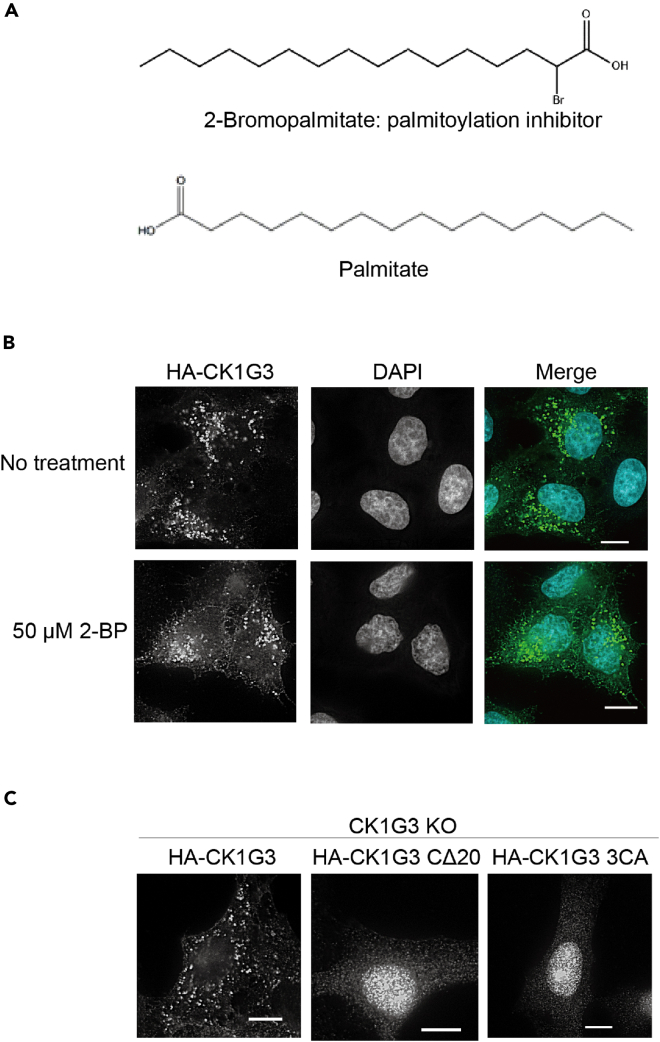
A palmitoylation inhibitor redistributes CK1G3 from the punctate compartments to the nucleocytoplasm (A) Chemical structures of 2-bromopalmitate and palmitate. (B) CK1G3 KO cells expressing HA-CK1G3 were cultured in a serum-free medium for 1 h and treated with 50 μM or without 2-bromopalmitate for 2 h at 37°C. Cells were fixed and immunostained with an anti-HA antibody. (C) Amino acid substitutions at the three cysteines in the *C*-terminal region abolished the endomembrane localization of CK1G3. The three cysteine residues (underlined in Figure 3C) that were candidates for palmitoylation sites were substituted with alanine residues in the CK1G3 3CA mutant. HA-CK1G3 3CA expressed in CK1G3 KO cells were immunostained with an anti-HA antibody. The scale bars indicated in microscopy images represent 10 μm.

Palmitoylation of CK1Gs has been identified in multiple palmitoyl-proteomics studies (Collins et al., 2017; Kang et al., 2008; Yang et al., 2010). A human palmitoyl-proteomics study has listed CK1G3 as a high-confidence candidate and CK1G1 and CK1G2 as medium-confidence candidates (Yang et al., 2010). Because palmitoylation influences the subcellular localization of various proteins (Fukata et al., 2016), we tested whether a palmitoylation inhibitor, 2-bromopalmitate (2-BP, Figure 6A), affected the localization of CK1G3. When cells were treated with 50 μM 2-BP, the endomembrane localized CK1G3s were partially diffused in the cytosol (Figure 6B). These results suggested that palmitoylation was involved in the localization of CK1G3 to punctate compartments, although we were unable to eliminate the possibility that the effect of 2-BP was not necessarily owing to the inhibition of protein palmitoylation (Davda et al., 2013).

Three cysteine residues conserved in the *C*-terminal regions of CK1G1, 2, and three are predicted to undergo the *S*-palmitoyl modification in the CSS-Palm program (Figure S3), which were absent in the CK1G3 CΔ20 mutant (Figure 3C). To examine whether these three cysteine residues expected to be palmitoylated are required for endomembrane localization of CK1G3, we generated a CK1G3 mutant construct (CK1G3 3CA), in which the three cysteine residues were replaced with alanine residues, and examined their subcellular localization by indirect immunofluorescence microscopy. Similar to CK1G3 CΔ20, CK1G3 3CA was localized to the cytosol and the nucleus (Figure 6C), which indicated the significance of the three cysteine residues in the endomembrane localization of CK1G3.

### The conserved *C*-terminal region is the key to determine the subcellular localization of CK1G family members

**Figure 7:**
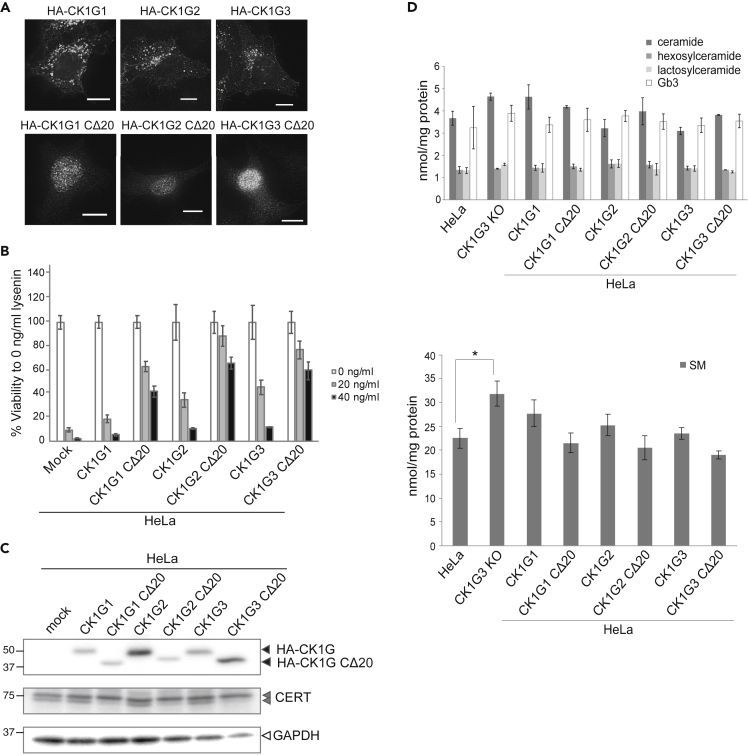
The conserved *C*-terminal region of the CK1G subfamily members is crucial for their subcellular distribution and function (A) Wild-type and CΔ20 mutants of CK1G1, 2, and 3 were expressed in HeLa cells and immunostained with an anti-HA antibody. The scale bars indicated in microscopy images represent 10 μm. (B) Lysenin sensitivity assay. Cells were seeded in 12-well plates, cultured overnight, and treated with 0, 20, or 40 ng/mL lysenin for 2 h at 37°C. Cell survival was measured by MTT assay. Results shown are the mean and SEM of three experiments. (C) *C*-terminus-deleted mutants of CK1G subfamily members were highly active at phosphorylating the CERT SRM. Wild-type CK1G members and their *C*-terminus-deleted mutants with an *N*-terminal HA-tag were expressed in HeLa cells. Lysate fractions prepared from the cells were analyzed by Western blotting with anti-HA antibody, anti-CERT (for the detection of endogenous CERT), and anti-GAPDH (as a loading control). (D) Sphingolipid analysis. Total lipids were extracted from the cells indicated and levels of the indicated sphingolipid species were quantified using an LC-MS/MS system. The amounts of the sphingolipid species were presented as bar graphs. Significance was determined using Dunnett’s test. The asterisk (∗) indicates a statistical difference at a five percent level. Results shown are the mean and SEM of three experiments.

The *C*-terminal region of CK1G1, 2, and 3 is highly conserved (Figure 1C), suggesting that the regulatory role for cellular localization may be common to the family members. We mildly overexpressed wild-type and CΔ20 mutants of CK1G1, 2, and 3 in HeLa cells and examined their cellular localization. As expected, CK1G1/2 and CK1G1/2 CΔ20 showed a similar localization pattern to CK1G3 and CK1G3 CΔ20, respectively (Figure 7A). HeLa cells overexpressing CK1G1, 2, and 3 gained a slight but a discernible resistance to lysenin, whereas overexpression of CΔ20 mutants conferred a more marked resistance (Figure 7B). Similar to the phenotype of CK1G3 CΔ20 expressing cells, the hyperphosphorylated form of endogenous CERT was more abundant in CK1G1/2 CΔ20 expressing cells, compared with CK1G1/2 expressing cells (Figure 7C).

### Effects of CK1G3 KO and overexpression of the CK1G CΔ20 mutants on the sphingolipidome

To examine whether CK1G KO affects the quantities of sphingolipids in cells, we conducted a lipidomic analysis of CK1G3 KO cells and the parental HeLa cells: Total lipid fractions extracted from the cells were subjected to LC-MS/MS to quantify the levels of ceramide, hexosylceramide, lactosylceramide, Gb3, and SM. The total cellular quantity of SM in CK1G3 KO cells was significantly increased compared with the quantity in HeLa cells (Figure 7D and Table S3), in line with previous studies showing that CK1G3 is quantitively the major CK1G isotype in HeLa cells (Itzhak et al., 2016) and that the CK1G-dependent hyperphosphorylation of the CERT SRM down-regulates the *de novo* synthesis of SM (Tomishige et al., 2009). We next tested the possibility that CK1G isotype-specific effects on the sphingolipidome might be detected when the wild-type or the CΔ20 mutants of CK1G1, 2, and 3 were overexpressed. Despite a significant decline in the *de novo* synthesis of SM in CK1G3ΔC cells (Figure 2C), we were unable to detect significant differences in the total cellular quantities of sphingolipids among the cells overexpressing wild-type or the CK1G3 CΔ20 mutant and the parental HeLa cells (Figure 7D and Table S3), presumably owing to a technical limitation, as discussed later in discussion.

## Discussion

**Figure 8:**
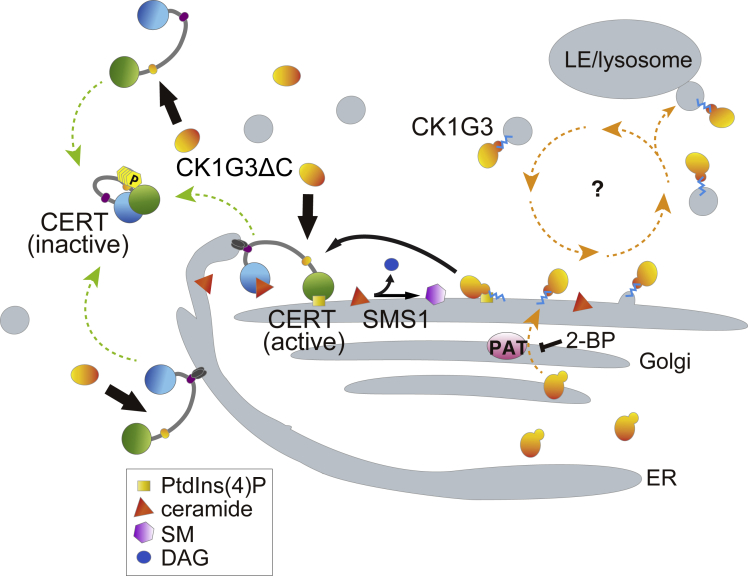
Compartmentalization-dependent functional control of CK1Gs After newly synthesized as a cytosolic protein, CK1G presumably binds to the Golgi-membrane via the electrostatic interaction between its *C*-terminal basic amino acids stretch and the Golgi localizing PtdIns(4)P (see also the text) and subsequently palmitoylated by the Golgi-residing palmitoyl acyltransferase (PAT). Palmitoylated CK1G is largely distributed to post-Golgi compartments and only partly to the Golgi apparatus, where SM synthase 1 (SMS1) is localized. CERT is associated with the ER via VAP-binding and with the Golgi via PtdIns(4)P-binding. It remains elusive whether palmitoylated CK1Gs are recycled among the distal- and post-Golgi compartments or eventually directed to lysosomes (for degradation) or exosomes (for secretion). The Golgi-distributed CK1G is spatially limited to interact only with CERT recruited to the ER-Golgi contact zone. In contrast, the *C*-terminus deleted CK1Gs are distributed throughout the cytosol and can phosphorylate CERT anywhere in the cells once the priming phosphorylation of CERT S132 by PKD occurs.

It has been poorly understood how the function of CK1Gs is regulated although the CK1Gs are house-keeping serine/threonine kinases regulating various fundamental events in cells (Knippschild et al., 2014). A previous study showed that CK1G regulates CERT activity via the phosphorylation of the SRM (Tomishige et al., 2009). Herein, we demonstrated that the *C*-terminal region of the CK1G family kinases is required for the retention of the kinase in the cytoplasmic punctate compartments probably through its modification by palmitoylation. A previous study using *Xenopus* showed that *C*-terminal deletion of CK1G1 does not affect its kinase activity but disturbs its plasma membrane localization and prevents Wnt signaling from reaching the cytoplasm (Davidson et al., 2005). The *C*-terminal 20 amino acid regions of human CK1Gs, particularly CK1G2 and CK1G3, are highly conserved (Figures 1C and 3C), and deletion of these regions similarly affected the intracellular localization of the kinases (Figure 7A). Palmitoylation of the three conserved cysteine residues within the *C*-terminal region is a strong candidate for the regulatory mechanism in the context of localization (Figures 6B and 6C) (Yang et al., 2010). In addition, a recent study suggested that basic amino acid residues present in the vicinity of palmitoylation sites of soluble *S*-acylated proteins are crucial for electrostatic interaction with PtdIns(4)P embedded in the Golgi membrane and for palmitoylation at the Golgi (Chumpen Ramirez et al., 2020). A basic amino acid stretch including nine lysine and arginine residues lies downstream of the cysteine residues in the human CK1G proteins (Figure 3C). These previous and current studies suggest that, after *de novo* synthesis as a cytosolic protein, CK1G binds to the Golgi membrane via the electrostatic interaction between its *C*-terminal basic stretch and the Golgi localizing PtdIns(4)P and subsequently is palmitoylated by a protein acyltransferase(s) to more firmly associate with membranes (Figure 8). Palmitoylated CK1Gs were largely distributed to punctate compartments (Figures 4 and 7A), which we assigned as distal- and post-Golgi compartments in this study, although we did not rule out the possibility that the CK1G-positive punctate compartments are other unknown or poorly characterized compartments. Deletion of the *C*-terminal region of CK1Gs abolished its retention in the punctate compartments (Figures 5 and 7A), which caused the SRM of the whole cellular CERT pool to be hyperphosphorylated (Figures 3B, 3D, and 7C). From these results, we propose a compartmentalization-based regulatory mechanism for CK1Gs, by which they access their substrates at specific intracellular organelles (Figure 8). In our current model, the palmitoylated CK1G is distributed largely to the post-Golgi compartments and only partly to the distal Golgi compartments, in which the palmitoylated CK1G can phosphorylate the SRM of CERT that is acting at the ER-Golgi contact sites. A previous study showed that the hyperphosphorylation of the CERT SRM lowers the affinity of the CERT PH domain for PtdIns(4)P (Sugiki et al., 2018). Hence, upon the SRM phosphorylation by the CK1Gs, CERT detaches from the Golgi apparatus, which may account for the observation that the colocalization of CK1G3 with CERT was less detectable, compared with the colocalization of CK1G3 with TGN46 (Figures 4 and 5A). In contrast to the palmitoylated form, non-palmitoylated CK1G is distributed throughout the nucleocytoplasm and can phosphorylate CERT anywhere in the cell (i.e., cytosol, ER, Golgi apparatus, and ER-Golgi contact sites). It remains unclear whether palmitoylated CK1Gs are recycled among the different compartments or eventually directed to lysosomes (for degradation) or exosomes (for secretion).

The synthesis of SM generates diacylglycerol (DAG) as a by-product. In addition, DAG acts as an activator of PKD (Baron and Malhotra, 2002; Pusapati et al., 2010), which phosphorylates S132 of CERT (Fugmann et al., 2007), triggering the CK1G-mediated sequential phosphorylation of the SRM (Tomishige et al., 2009). Recent studies have shown that “stimulator of interferon genes” (STING), a membrane-spanning protein, is localized to SM- and cholesterol-enriched subdomains in the TGN, depending on its palmitoylation (Mukai et al., 2016; Takahashi et al., 2021). By analogy, it is conceivable that the probability of the retention of palmitoylated CK1Gs in the distal Golgi compartments is increased as the SM level in the Golgi increases, thereby enhancing the phosphorylation of the SRM of CERT recruited to the ER-Golgi contact zone. The ER-to-Golgi transport of ceramide by CERT may be delicately tuned by a complicated system comprising various proteins, such as VAP, PtdIns(4)P-kinase beta (PI4KB), CK1G, PKD, and SMS; and lipids, such as PtdIns(4)P, SM, and DAG, that are distributed to the ER-Golgi contact zones as we previously hypothesized (Goto et al., 2020).

Not only was the GFP-lysenin binding level reduced but so was the *de novo* synthesis level of SM in CK1G3ΔC cells, compared with the wild-type control levels (Figures 2B and 2C), indicating that CK1G3 is involved in the regulation of the *de novo* synthesis of SM directed to the PM. Nevertheless, we were unable to detect a clear reduction in the total cell-associated SM levels in the CΔ20 mutants of CK1G1, 2, and 3 (Figures 7D and Table S3). This is probably owing to a technical limitation, in that standard MS-based lipidomics is unable to distinguish *de novo* synthesized SM from serum-derived endocytosed SM, although following endocytosis, lipoprotein-associated SM is largely destined to enter the degradation pathway and not to be utilized *en bloc* as membrane SM (Chatelut et al., 1998). Indeed, a previous study showed that prolonged culture under serum-depleted conditions is required to exhibit a clear reduction in the total cell-associated SM levels in SM synthesis-deficient mutant cells (Tachida et al., 2020). The amino acid sequences of the kinase domain are highly conserved among the three CK1G subtypes, while their *N*-terminal and *C*-terminal regions, which may determine their cellular functions (Han et al., 2014), are unique to each subtype (Figure 1C). To examine CK1G subtype-specific impacts on the lipidome in cells, the use of serum-free cell culture conditions may be required.

Several independent human genetic studies have recently shown that *de novo* mutations in *CERT1* (which encodes CERT) induce inherited intellectual disorders (de Ligt et al., 2012; Deciphering Developmental Disorders Study, 2015; Hamdan et al., 2014; Lelieveld et al., 2017; Tamura et al., 2021). Among intellectual disorder-related variants in *CERT1*, many, but not all, variants are mapped to the region encoding the SRM of CERT (Murakami et al., 2020). These human disorder-associated variants in the CERT SRM have been experimentally shown to impair the SRM phosphorylation-dependent downregulatory mechanism and to render the mutated CERT abnormally active (Murakami et al., 2020). Intriguingly, a recent study reported that *de novo* variants in *CSNK1G1* were associated with syndromic developmental delay and autism spectrum disorder in humans (Gold et al., 2020). Three individuals had variants within the kinase domain of CK1G1, and two individuals had variants within the *C*-terminal region. The kinase-dead mutation might partly impair the hyperphosphorylation of CERT. In contrast, if a variant located in the vicinity of the three cysteine residues, which is predicted to disrupt splicing, affects the subcellular localization of CK1G1, the *C*-terminus mutated CK1G1 might act as a hyperactive kinase, which may constitutively down-regulate the function of CERT as discussed above. If this were the case, inappropriate down-regulation of CERT by hyperactive types of *CSNK1G* mutations might also cause the central neuron system-related disorders. In this context, chemical inhibitors and activators of CK1G might be applicable to a new pharmaceutical in the future. However, pertaining to CK1G3 inhibitors as a target to manipulate ceramide levels in cells, we consider that this application direction is impractical because modulation (inhibition and activation) of the housekeeping kinase CK1Gs would impact various cellular evens in addition to ceramide transfer, which should cause various side effects. For the aim to manipulate ceramide levels in cells, modulators specific to the ceramide metabolism-dedicated proteins such as CERT and the ceramide synthases CerSs should be more suitable. Cells seem to regulate the metabolism of ceramide and SM in a much more complicated and elegant manner than we imagined, and further studies are needed to address these new hypotheses.

In conclusion, genome-wide gene disruption screening with an SM-binding cytolysin allowed us to find that loss of the *C*-terminal region of CK1G3 rendered the kinase hyperactive in cells. Further analyses revealed that the *C*-terminal region of CK1G including cysteine residues expected to be palmitoylated sites is a determinant to localize the kinase presumably to the distal- and post-Golgi compartments. The compartmentalization of CK1G is crucial for the regulatory phosphorylation of the SRM of CERT and loss of the compartmentalization causes constitutive hyperphosphorylation of the CERT SRM, which inactivates CERT and consequently down-regulates *de novo* SM synthesis.

### Limitations of the study

The current study revealed that the *C*-terminal region of the CK1G family of kinases determines their subcellular compartmentalization and plays a crucial role in controlling the phosphorylation state of CERT, which in turn regulates the synthesis of SM. Palmitoylation at the cysteine residues in the *C*-terminal region of CK1Gs redistributes the kinases to punctate compartments, which partially overlap with the distal- and post-Golgi compartments, although the precise identity of these compartments remains unclear. In addition, how the palmitoylation of CK1Gs is regulated remains unknown. Further investigation of the regulatory mechanism of CK1G activity is expected to provide much insight into the regulation of the intracellular trafficking of ceramide and synthesis of SM.

## STAR★Methods

### Key resources table

**Table:** 

REAGENT or RESOURCE	SOURCE	IDENTIFIER
**Antibodies**		

Anti-HA	Roche Diagnostics	Cat#3F10; RRID: AB_390919
anti-CERT	Abcam	Cat#: ab72536; RRID: AB_2082802
chicken antibody against VAP	Kumagai et al., 2014	N/A
anti-TGOLN2/TGN46	Bethyl Laboratories	Cat#: A304-434A; RRID: AB_2620628
anti-TOMM20	Sigma-Aldrich	Cat#: WH0009804M1; RRID: AB_1843992
anti-Lamp2	Santa Cruz	Cat#: sc-18822; RRID: AB_626858
anti-LBPA	Echelon Bioscience	Cat#: z-PLBPA; RRID: AB_11129226
anti-EEA1	BD Transduction Laboratories	Cat#: 610457; RRID: AB_397830
anti-catalase	Cell Signaling Technology	Cat#: 12980; RRID: AB_2798079
anti-Rab11a	Cell Signaling Technology	Cat#: 2413; RRID: AB_2173452
anti-Hrs	Proteintech	Cat#: 10390-1-AP; RRID: AB_2118914
anti-GAPDH	Fujifilm Wako Pure Chemical Corporation	Cat#: 016-25523; RRID: AB_2814991
Anti-GFP	Nacalai Tesque	Cat#: 04404-84; RRID: AB_10013361

**Chemicals, peptides, and recombinant proteins**		

PPI-2	Merck Millipore	P5726
PPI-3	Merck Millipore	P0044
EDTA-free protease inhibitor cocktail	Roche Diagnostics	11836170001
λ protein phosphatase	New England Biolabs	P0753S
2-Bromohexadecanoic acid (2-BP)	Sigma-Aldrich	M1177
Lipid dye II	Dojindo Laboratories	LD02
Lysenin	Gift from Dr. Sekizawa (Zenyaku Kogyo)	N/A
GFP-lysenin	Gift from Dr. Kobayashi (RIKEN)	N/A
[^14^C(U)]L-serine	Moravec	MC265
Nutridoma-SP	Sigma-Aldrich	11011375001
MTT	Nacalai Tesque	23547-34
Mildform 10N	Fujifilm Wako Pure Chemical Corporation	133-10311

**Deposited data**		

Raw data	This study	https://doi.org/10.17632/cyp2x8cthd.1

**Experimental models: Organisms/strains**		

HeLa-mCAT#8 cells	Yamaji et al., 2010	N/A
CK1G3 KO cells	This study	N/A
CK1G3ΔC cells	This study	N/A

**Oligonucleotides**		

see Table S3	This study	N/A

**Software and algorithms**		

ATUM gRNA Design Tool	ATUM	N/A
SeaView BioNJ algorithm	Gouy et al., 2010	N/A
CSS-Palm	Ren et al., 2008	N/A

### Resource availability

#### Lead contact

Further information and requests for resources should be directed to and will be fulfilled by the Lead Contact, Kentaro Hanada (hanak@nih.go.jp).

#### Materials availability

Plasmids generated in this study will be provided on requests.

### Experimental model and subject details

#### Cell lines

HeLa-mCAT#8 cells (Yamaji et al., 2010) were cultured in DMEM with 10% (v/v) FBS at 37°C with 5% CO_2_.

### Method details

#### Antibodies and reagents

Anti-HA antibody (#3F10) was purchased from Roche Diagnostics; anti-CERT (#ab72536) from Abcam; chicken antibody against VAP (Kumagai et al., 2014) anti-TGOLN2/TGN46 (#A304-434A) from Bethyl Laboratories; anti-TOMM20 (#WH0009804M1) from Sigma-Aldrich; anti-Lamp2 (#sc-18822) from Santa Cruz; anti-LBPA (#z-PLBPA) from Echelon Bioscience; anti-EEA1 (#610457) from BD Transduction Laboratories; anti-catalase (#D4P7B) and anti-Rab11a (#2413) from Cell Signaling Technology; anti-Hrs (#10390-1-AP) from Proteintech; anti-GFP (#04404-84) from Nacalai Tesque, and anti-GAPDH (016-25523) from Fujifilm Wako Pure Chemical Corporation. 2-Bromohexadecanoic acid (2-BP, #M1177) was purchased from Sigma-Aldrich. Lipid dye II (#LD02) was from Dojindo Laboratories. Lysenin was a gift from Dr. Sekizawa (Zenyaku Kogyo). GFP-lysenin was a gift from Dr. Kobayashi (Riken). [^14^C(U)]L-serine (156 mCi/mmol, #MC265) was from Moravec.

#### Isolation of *CK1G3*, and plasmid constructions

The open reading frame (ORF) of *CK1G3* was amplified by PCR using total cDNA from HeLa cells as a template and cloned into the pBSnHAcFL vector (Kawano et al., 2006). The *N*-terminally HA-tagged *CK1G3* was subcloned into the pMXs-IRES-Neo vector (Cell Biolabs Inc.) or pcDNA3.1(+) vector (Invitrogen) for expression in mammalian cells. The ORFs of *CK1G* and *CK1G2* were amplified by PCR using the HA-CK1G1/pCMV3 plasmid (Sino Biological) and the HA-CK1G2/pMXs-IRES-IN plasmid (Tomishige et al., 2009) as templates, respectively, and cloned into the pMXs-IRES-Neo vector (Cell Biolabs Inc.) for expression in mammalian cells. To prevent cellular toxicity, CK1G2 was expressed with its 5’ untranslated region as described in our previous study (Tomishige et al., 2009). Site directed mutagenesis was performed using the PrimeSTAR mix (TaKaRa). Plasmids for genome editing were constructed by ligating cDNAs that encode single-guide RNA (sgRNA) sequences into the pSELECT-CRISPR-Cas9 vector (Ogawa et al., 2018; Yamaji et al., 2019). SgRNA sequences were designed using the ATUM CRISPR gRNA design tool (https://www.atum.bio/catalog/vectors/grna-design). Primers and cloning strategies used for constructing all the plasmids used in this study are summarized in Table S4.

#### Genome editing in HeLa cells

Genome editing was performed as described elsewhere (Yamaji et al., 2019). Briefly, the SgRNAs *CSNK1G3*-ex12 and *CSNK1G3*-ex5, which individually recognize the sequences in exon 12 and exon 5, were designed. HeLa-mCAT#8 cells were transfected with CRISPR plasmids in a 12-well plate and cultured overnight. The next day, cells were trypsinized, transferred to a 6-well plate, and selected with 0.5 μg/ml puromycin for two to three days. After selection, cells were cultured in puromycin-free media for up to one week until cell numbers were sufficiently expanded for sample preparation for genome sequencing and lysenin sensitivity assays. Genome sequencing was performed to identify indels within the flanking regions of the sgRNA targeting sequences. Briefly, cells were trypsinized, heated in TE buffer, and vortexed. After centrifugation, the supernatant was collected and used as a template for genomic PCR. The PCR products were directly sequenced, and clones with frameshift mutations in all alleles were further analyzed.

#### Metabolic labelling of lipids

Metabolic labelling of sphingolipids with radioactive serine was performed as described previously with some modifications (Fukasawa et al., 1999). Briefly, cells seeded in 6 well plates were cultured overnight at 37°C, washed once with serum-free DMEM, and 1 ml of DMEM containing 1% Nutridoma-SP (Roche) and 9.25 kBq of L-[U-^14^C] serine (Moravek Inc., #MC-265) was added to each well. After metabolic labelling for 24 h at 37°C, cells were washed twice with ice-cold PBS, and lysed in ice-cold water containing 0.1% SDS. The lysates were transferred to lock-tubes and sonicated in wet ice until the lysates become smooth. Protein concentration was determined by BCA assay (Thermo Fisher Scientific), and an equivalent amount of each sample was subjected to lipid extraction using Bligh and Dyer method (Bligh and Dyer, 1959). The extracted lipids were separated on TLC plates (Millipore) with solvent of methyl acetate/*n*-propanol/chloroform/methanol/025% potassium chloride (25:25:25:10:9) (Hanada et al., 1997). The resulting TLC plates were air-dried and exposed to imaging plates for 7 days. Images were acquired by an image analyzer (Typhoon FLA 7000, GE Healthcare), and the signal intensity of the bands corresponding to SM were quantified using an analysis software (ImageQuant TL, GE Healthcare).

#### MTT assay

Cells seeded in 24 well plates were cultured overnight at 37°C, washed once with serum-free DMEM and treated with various concentration of lysenin (0, 20, 40, 80, 160, 320 ng/ml) diluted in serum free DMEM for 2 h at 37°C. The media was discarded, and MTT solution [1:1 ratio mixture of serum-free DMEM and 5mg/ml MTT (Nacalai Tesque) resolved in PBS] was added and incubated for 30 min at room temperature with protection from light. The MTT solution was discarded, and lysis solution (4 mM HCl, 0.1% NP40 in isopropanol) was added and incubated for 30 min at room temperature with protection from light. The cell lysate was transferred to a 96 well plate, and absorbance at λ595 was measured using iMark Microplate Reader (BIORAD).

#### Immunofluorescence microscopy and flow cytometry

Cells cultured on glass cover slips were fixed in Mild-form 10N (Fujifilm Wako Pure Chemical Corporation) at room temperature for 15 min, permeabilized in 0.1% (v/v) Triton X-100/PBS at 4°C for 20 min, blocked in 3% (v/v) BSA/PBS, and incubated with primary and secondary antibodies diluted in 0.1% (v/v) BSA/PBS. The cover slips were rinsed with distilled water and mounted in Fluoromount (Diagnostic BioSystems). A fluorescence microscope (BZ-X710, Keyence Corp.) with a 60x Plan-Apochromat V NA 1.20 objective lens was used for fluorescence imaging. All of the scale bars indicated in microscopy images represent 10 μm.

For flow cytometry, cells harvested after trypsinization were washed with PBS and then with 1% (w/v) BSA/PBS. The washed cells were re-suspended in 1% (w/v) BSA/PBS with or without GFP-lysenin (final 15 μg/mL) and incubated on ice for 1 h. After washing with 1% (w/v) BSA/PBS, cells were passed through a mesh filter and subjected to analysis with the FACSCalibur™ (BD BioScience).

#### λ protein phosphatase treatment

Cells were grown in 6-well plates, lysed in 1% (v/v) Triton X-100/TBS containing the cOmplete™ EDTA-free protease inhibitor cocktail (Roche Diagnostics) on ice for 10 min, centrifuged at 20,400 × *g* for 10 min, and the supernatant was subjected to λ Protein phosphatase treatment. For negative control samples, protein phosphatase inhibitor solutions (PPI-2 and PPI-3, Merck Millipore) were added to the lysis buffer. λ Protein phosphatase treatment was performed according to the manufacturer’s instructions. Briefly, 200 units of λ protein phosphatase (New England BioLabs, Inc.) was added to the cell lysate containing 30 μg of total protein and incubated at 30°C for 15 min.

#### Sphingolipid analysis

Cells were seeded in a 6-cm dish at a density of 0.5 × 10^6^ cells/dish in 5 mL of culture medium. After 24 h, cells were washed twice with PBS and harvested. The amount of protein was determined and lipids were extracted from the cells. A total of 1 nmol each of the internal standards C17:0 SM, C17:0 ceramide (Cer), C17:0 lactosylceramide, C17:0 glucosylceramide, C17:0 Gb3, and d18:1-d5-C18:0 GM3 (Avanti Polar Lipids, Inc) were then added for quantification. Sphingolipids were analyzed by an LC-MS/MS system that consisted of a Prominence UFLC system (Shimadzu Corporation) coupled to a 3200 QTRAP System (SCIEX) as described previously (Nakao et al., 2019).

### Quantification and statistical analysis

The two-tailed *t*-test was used to assess differences for *de novo* SM synthesis and hyperphospho/de-/hypo-phopho CERT levels of the genome edited HeLa cells. The Dunnett’s test was used to assess differences for SM levels in the sphingolipid analysis. p values were consiered statistically significant if lower than 0.05. All experiments were repeated with a minimum of three independent experiments. Mean and standard error of the mean (SEM) is shown in all figures unless stated. Statistical analysis was performed using Microsoft Excel for Mac (Microsoft Corporation).

## Data Availability

• Raw data have been deposited at Mendeley Data and are publicly available as of the date of publication. Accession numbers are listed in the key resources table. • This paper does not report original code. • Any additional information required to reanalyze the data reported in this paper is available from the lead contact upon request.
