# Supplementary material for: Compartmentalization of casein kinase 1 γ CSNK1G controls the intracellular trafficking of ceramide
Source: iScience. 2022 Jun 16;25(7):104624. doi: 10.1016/j.isci.2022.104624 (PMC9254030; doi:10.1016/j.isci.2022.104624)
Supplement: Document S1. Figures S1–S3 and Tables S1–S4 [file mmc1.pdf]

iScience, Volume *25*

Supplemental information

Compartmentalization of casein kinase 1 γ CSNK1G controls the intracellular trafficking of ceramide

Asako Goto, Shota Sakai, Aya Mizuike, Toshiyuki Yamaji, and Kentaro Hanada

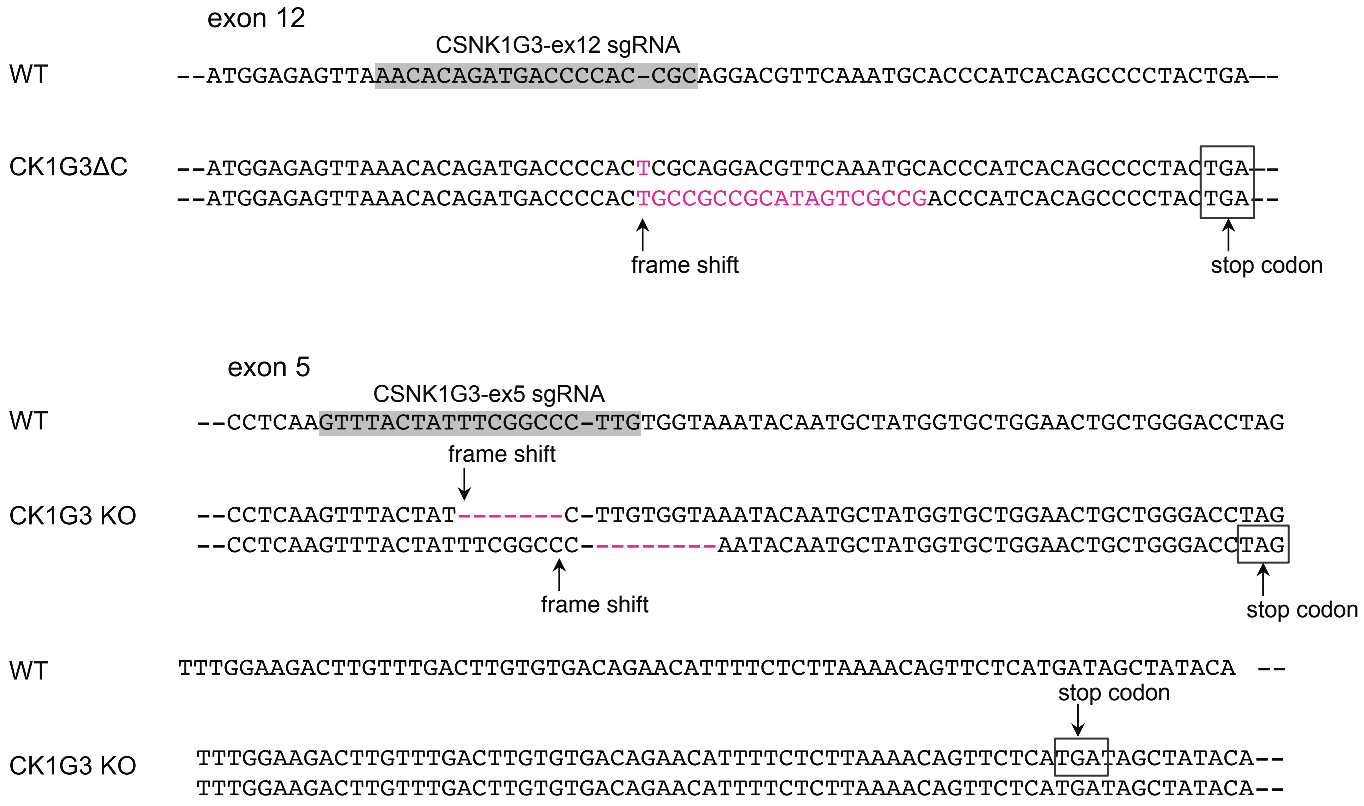

Figure S1. Construction of CSNK1G3 mutant HeLa cells by genome editing (related to Figure 1C). Nucleotide sequences corresponding to the sgRNAs are highlighted with gray shading. Nucleotides deleted or inserted by genome editing are shown in magenta. Positions of frame shifts and stop codons arising from genome editing are indicated.

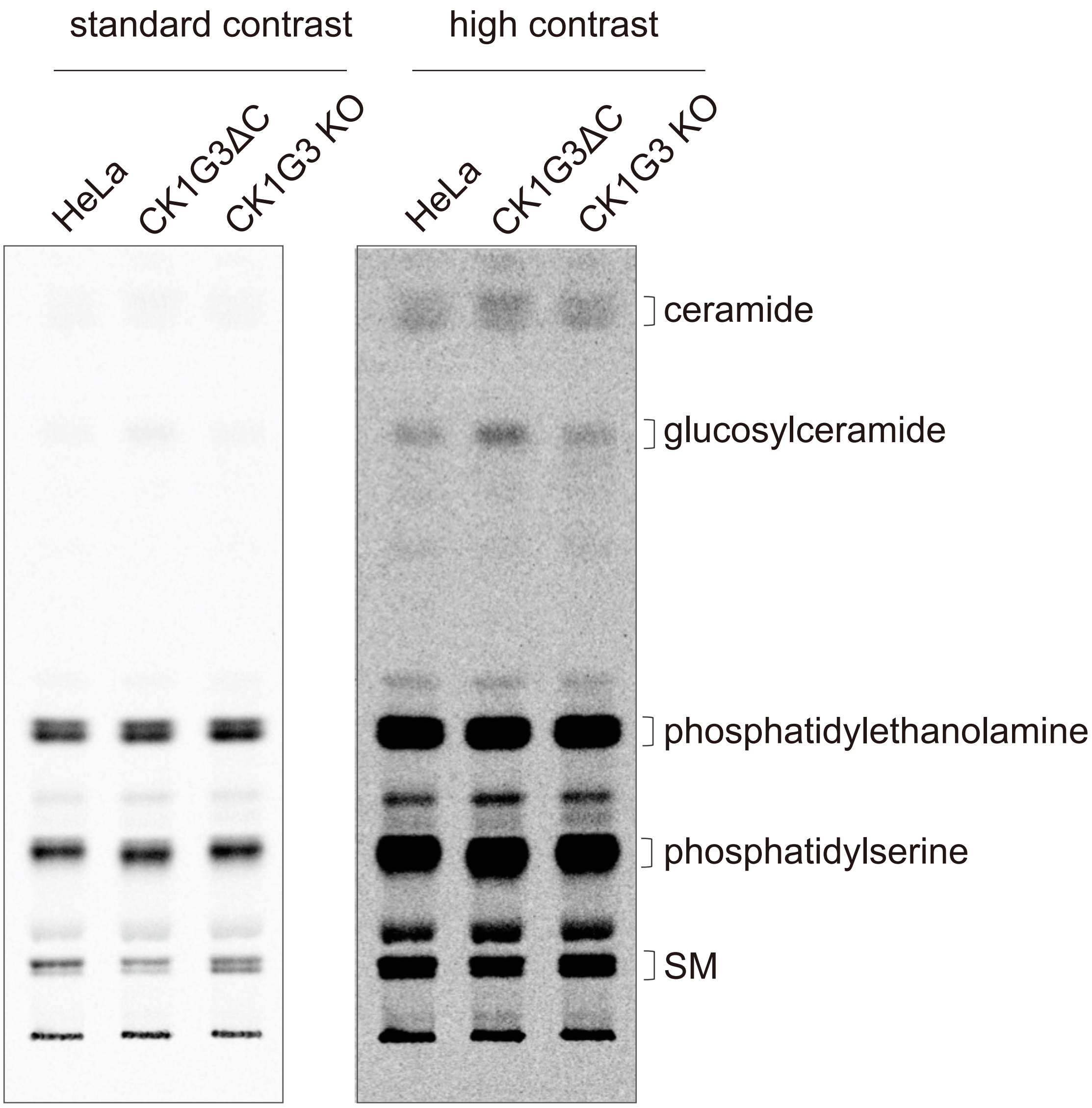

Figure S2. Standard and high contrast TLC images of metabolic labeling of lipids with radioactive serine in various HeLa cell lines (related to Figure 2C).
De novo synthesis of SM was measured by metabolic labeling of lipids with [14C]serine for 24 h. Lipid fractions extracted from HeLa, CK1G3ΔC, and CK1G3 KO cells were separated by TLC, and radioactive lipids separated on the TLC plate were visualized with an image analyzer. The obtained standard contrast image was used to quantify the amount of de novo synthesized SM. For clear visualization of radioactive ceramide and glucosylceramide, a high contrast image made with the Photoshop program is also shown.

ID	Position	Peptide	Score	Cutoff
Homo_sapiens_CK1G1	406	EVVEEAKCCCFFKRK	8.667	3.419
Homo_sapiens_CK1G1	407	VVEEAKCCCFFKRKR	8.428	3.419
Homo_sapiens_CK1G1	408	VEEAKCCCFFKRKRK	6.563	4.222

ID	Position	Peptide	Score	Cutoff
Homo_sapiens_CK1G2	399	EVADETKCCCFFKRR	7.626	3.419
Homo_sapiens_CK1G2	400	VADETKCCCFFKRRK	8.134	3.419
Homo_sapiens_CK1G2	401	ADETKCCCFFKRRKR	6.287	4.222

ID	Position	Peptide	Score	Cutoff
Homo_sapiens_CK1G3	431	EVMDETKCCCFFKRR	7.826	3.419
Homo_sapiens_CK1G3	432	VMDETKCCCFFKRRK	7.406	3.419
Homo_sapiens_CK1G3	433	MDETKCCCFFKRRKR	6.722	4.222

Figure S3. Prediction of palmitoylation sites in CK1G3 (related to Figure 3C).
Palmitoylation sites in CK1G predicted using the CSS-Palm program (Ren et al., 2008) are shown. For the prediction, the full amino acid sequences of CK1G1/2/3 were used as a query under a high stringency threshold, and the highest values of the candidacy score were assigned to the three cysteine residues in the C-terminal regions of CK1G1/2/3.

Table S1. List of accession numbers of the protein sequences used for phylogenetic analysis (related to Figure 1A)

Organism	Protein name	Accession number
Homo Sapiens	CK1G1	GenBank: NP_001316535
Homo Sapiens	CK1G2	GenBank: NP_001310
Homo Sapiens	CK1G3	GenBank: NP_004375
Oryctolagus cuniculus	CK1G1	GenBank: XP_002718169
Oryctolagus cuniculus	CK1G3	GenBank: XP_002710201
Mus musculus	CK1G1	GenBank: NP_775277
Mus musculus	CK1G2	GenBank: NP_001153063
Mus musculus	CK1G3	GenBank: NP_690022
Molossus molossus	CK1G1	GenBank: KAF6499645
Molossus molossus	CK1G2	GenBank: XP_036137908
Molossus molossus	CK1G3	GenBank: XP_036134729
Balaenoptera musculus	CK1G1	GenBank: XP_036697503
Balaenoptera musculus	CK1G2	GenBank: XP_036703105
Balaenoptera musculus	CK1G3	GenBank: XP_036703031
Xenopus leavis	CK1G1	GenBank: NP_001084836
Xenopus leavis	CK1G2	GenBank: NP_001086018
Xenopus leavis	CK1G3	GenBank: NP_001089290
Chelonia mydas	CK1G isoform X2	GenBank: XP_037767502
Chelonia mydas	CK1G isoform X10	GenBank: XP_037757563
Bactrocera dorsalis	CK1G isoform X2	GenBank: XP_011204591
Gekko japonicus	CK1G2	GenBank: XP_015272641
Gekko japonicus	CK1G3	GenBank: XP_015271704
Danio rerio	CK1G1	GenBank: NP_001008635
Danio rerio	CK1G2	GenBank: XP_005171263
Danio rerio	CK1G3	GenBank: NP_001039315
Gallus gallus	CK1G1	GenBank: NP_001005800
Gallus gallus	CK1G isoform X2	GenBank: XP_015155357
Phallusia mammillata	CK1G3	GenBank: CAB3233978

Table S2. *CSNK1G* sgRNAs included in libraries A and B

Gene name	Exon number	sgRNA sequence	Fold enrichment of sgRNA (lysenin treated/non-treated)			
			Llibrary A batch 1	Library A batch 2	Library B batch 1	Library B batch 2
CSNK1G1	2	GCACACTGCTCTCGACCATC	0.9	0.9	NI	NI
CSNK1G1	2	ATCGTCCTCTGGGGTTCTTA	0.0	0.0	NI	NI
CSNK1G1	4	CTGTACTCTAAATGAAGCTG	1.4	0.2	NI	NI
CSNK1G2	5	TCTACTACTTCGGTCCGTGC	0.2	0.6	NI	NI
CSNK1G2	5	TCCGGTCGCACAGGTCGAAC	0.6	0.2	NI	NI
CSNK1G2	6	GCTTCACGTCCCGGTAGATT	0.4	0.2	NI	NI
CSNK1G3	6	CGTCCTATTAAGAAGTTCTC	0.0	0.0	NI	NI
CSNK1G3	12	GGGTGCATTTGAACGTCCTG	22.0	9.6	NI	NI
CSNK1G3	1	TTAAACCAAGATTTCTCGCA	0.1	0.1	NI	NI
CSNK1G1	3	TGAATATGTAGCAATCAAAC	NI*	NI	0.8	0.0
CSNK1G1	3	TTTGATTGCTACATATTCAT	NI	NI	1.1	0.3
CSNK1G1	2	GGATCATGGACCATCCTAGT	NI	NI	1.0	0.9
CSNK1G2	6	CCCCCTGCAGATCACGCGCA	NI	NI	0.2	0.0
CSNK1G2	4	TACAAGCAGCTCAGCGCCAC	NI	NI	0.3	0.1
CSNK1G2	4	CCCAGGAGCCGATCAAGTCC	NI	NI	0.8	0.0
CSNK1G3	7	AGAGACGATTTAGAAGCTTT	NI	NI	0.2	0.0
CSNK1G3	12	AACACAGATGACCCCACCGC	NI	NI	16.1	15.2
CSNK1G3	7	ATTACCTTTAAGCCTTGCCA	NI	NI	0.0	0.4

* NI: The sgRNA is not included in the library which is shown in the top of the line.

Exon numbers are based on the sequences of the longest transcript variants deposited in the Aceview database (https://www.ncbi.nlm.nih.gov/IEB/Research/Acembly/).

Table S3 Sphingolipid levels in HeLa cells with altered CK1G expression levels (related to Figure 7D)

Sphingolipid species	Fatty acyl chain length	HeLa		CK1G3 KO		HeLa/CK1G1		HeLa/CK1G1 C⊿20		HeLa/CK1G2		HeLa/CK1G2 C⊿20		HeLa/CK1G3		HeLa/CK1G3 C⊿20	
		lipid mass (nmol/mg protein)	SEM	lipid mass (nmol/mg protein)	SEM	lipid mass (nmol/mg protein)	SEM	lipid mass (nmol/mg protein)	SEM	lipid mass (nmol/mg protein)	SEM	lipid mass (nmol/mg protein)	SEM	lipid mass (nmol/mg protein)	SEM	lipid mass (nmol/mg protein)	SEM
Ceramide	d18:1-C16:0	1.06	0.09	1.90	0.09	1.36	0.19	1.24	0.08	0.75	0.10	1.06	0.18	0.79	0.10	1.02	0.05
	d18:1-C18:0	0.27	0.04	0.38	0.02	0.32	0.04	0.28	0.00	0.17	0.03	0.25	0.02	0.19	0.01	0.25	0.01
	d18:1-C20:0	0.11	0.01	0.14	0.02	0.11	0.00	0.10	0.00	0.08	0.02	0.10	0.01	0.09	0.01	0.11	0.01
	d18:1-C22:0	0.44	0.06	0.50	0.02	0.53	0.07	0.42	0.01	0.32	0.04	0.45	0.06	0.34	0.03	0.45	0.03
	d18:1-C24:1	1.21	0.10	1.19	0.14	1.60	0.16	1.53	0.08	1.34	0.15	1.47	0.33	1.17	0.08	1.37	0.02
	d18:1-C24:0	0.58	0.05	0.53	0.07	0.71	0.09	0.61	0.07	0.57	0.05	0.63	0.08	0.51	0.04	0.62	0.02
	total	3.67	0.32	4.64	0.16	4.63	0.55	4.18	0.05	3.22	0.38	3.97	0.61	3.09	0.17	3.82	0.02
HexosylCeramide	d18:1-C16:0	0.09	0.01	0.36	0.01	0.10	0.01	0.10	0.01	0.10	0.01	0.09	0.02	0.09	0.00	0.09	0.00
	d18:1-C18:0	0.03	0.00	0.06	0.00	0.02	0.00	0.02	0.00	0.02	0.00	0.02	0.00	0.02	0.00	0.02	0.00
	d18:1-C20:0	0.09	0.01	0.10	0.00	0.06	0.00	0.06	0.01	0.07	0.01	0.06	0.02	0.07	0.01	0.08	0.01
	d18:1-C22:0	0.26	0.03	0.24	0.01	0.21	0.02	0.23	0.02	0.26	0.03	0.24	0.04	0.23	0.01	0.24	0.02
	d18:1-C24:1	0.58	0.06	0.45	0.01	0.76	0.06	0.81	0.06	0.81	0.09	0.84	0.08	0.73	0.05	0.64	0.02
	d18:1-C24:0	0.31	0.05	0.19	0.01	0.29	0.02	0.29	0.02	0.35	0.04	0.32	0.00	0.28	0.03	0.28	0.01
	total	1.35	0.15	1.40	0.02	1.44	0.11	1.50	0.11	1.62	0.18	1.58	0.15	1.43	0.08	1.35	0.00
Sphingomyelin	d18:1-C16:0	7.14	0.70	16.35	1.76	8.51	0.95	6.72	0.70	7.65	0.62	6.50	0.90	7.05	0.19	6.35	0.10
	d18:1-C18:0	0.98	0.10	1.79	0.14	0.90	0.10	0.62	0.03	0.73	0.06	0.59	0.03	0.73	0.06	0.65	0.01
	d18:1-C20:0	0.28	0.03	0.50	0.06	0.21	0.03	0.17	0.01	0.19	0.04	0.15	0.02	0.20	0.01	0.16	0.01
	d18:1-C22:0	2.20	0.20	2.31	0.21	2.16	0.20	1.60	0.17	2.01	0.24	1.56	0.14	1.90	0.18	1.59	0.12
	d18:1-C23:0	0.42	0.05	0.48	0.03	0.46	0.05	0.37	0.03	0.45	0.05	0.37	0.04	0.41	0.04	0.34	0.02
	d18:1-C24:1	9.03	0.76	8.39	0.37	12.72	1.37	9.86	0.91	11.66	1.11	9.22	1.25	10.89	0.56	8.00	0.43
	d18:1-C24:0	2.47	0.27	2.02	0.17	2.74	0.20	2.17	0.22	2.55	0.20	2.14	0.18	2.38	0.21	1.93	0.16
	total	22.52	2.07	31.84	2.55	27.70	2.81	21.50	2.05	25.24	2.25	20.54	2.54	23.55	1.21	19.02	0.80
Lactosylceramide	d18:1-C16:0	0.14	0.01	0.44	0.00	0.16	0.02	0.15	0.01	0.16	0.02	0.13	0.02	0.13	0.00	0.14	0.00
	d18:1-C18:0	0.04	0.00	0.07	0.01	0.03	0.01	0.03	0.00	0.03	0.00	0.02	0.00	0.03	0.00	0.03	0.00
	d18:1-C20:0	0.14	0.01	0.15	0.01	0.09	0.02	0.08	0.00	0.09	0.00	0.08	0.01	0.08	0.01	0.09	0.01
	d18:1-C22:0	0.27	0.02	0.29	0.02	0.24	0.02	0.22	0.03	0.24	0.02	0.21	0.04	0.21	0.02	0.23	0.01
	d18:1-C24:1	0.49	0.06	0.46	0.00	0.67	0.11	0.65	0.03	0.80	0.12	0.69	0.15	0.72	0.07	0.56	0.02
	d18:1-C24:0	0.24	0.03	0.18	0.01	0.24	0.04	0.23	0.01	0.31	0.03	0.23	0.04	0.24	0.03	0.21	0.01

	total	1.31	0.13	1.59	0.05	1.43	0.18	1.35	0.07	1.62	0.18	1.37	0.26	1.41	0.12	1.25	0.04
Gb3	d18:1-C16:0	0.32	0.08	1.12	0.05	0.31	0.03	0.33	0.02	0.30	0.01	0.28	0.02	0.28	0.03	0.34	0.01
	d18:1-C18:0	0.10	0.03	0.21	0.03	0.06	0.01	0.07	0.00	0.07	0.00	0.06	0.00	0.05	0.00	0.07	0.01
	d18:1-C20:0	0.19	0.04	0.21	0.06	0.13	0.00	0.17	0.01	0.17	0.01	0.16	0.02	0.17	0.02	0.16	0.01
	d18:1-C22:0	0.42	0.17	0.52	0.12	0.38	0.05	0.42	0.09	0.44	0.02	0.40	0.03	0.38	0.05	0.48	0.03
	d18:1-C24:1	1.59	0.43	1.41	0.08	1.91	0.18	2.01	0.32	2.10	0.18	2.03	0.28	1.94	0.21	1.88	0.18
	d18:1-C24:0	0.61	0.22	0.42	0.05	0.58	0.08	0.60	0.07	0.68	0.03	0.59	0.02	0.53	0.06	0.60	0.06
	total	3.24	0.95	3.89	0.36	3.37	0.34	3.59	0.52	3.77	0.25	3.52	0.35	3.35	0.33	3.54	0.30

Results shown are the mean and SEM of three experiments.

Table S4 List of plasmids and primers used in this study (related to STAR methods)

**Plasmid**	**Source of inserted sequence**	**Primer set**	**Enzyme sites**	**Cloning method, identifier**
CSNK1G3-ex5/ pSELECT-CRISPR-Cas9	N/A	5'-CACCGTTTACTA TTTCGGCCCTTG-3'<br>5'-AAACCAAGGGCCGAAATAGTAAAC-3'	N/A	This study
CSNK1G3-ex12/ pSELECT-CRISPR-Cas9	N/A	5'-CACCGAACACAGATGACCCCACCGC-3'<br>5'-AAACGCGGTGGGGTCATCTGTGTTC-3'	N/A	This study
FLAG-CERT/pcDNA3.1(+)	N/A	N/A	N/A	Kawano et al., 2006
pMXs-IBU-hCERT-GS-Venus	N/A	5'-GGTGGTACGGGAATTCACCAAGCTTATGTCGGATAATC-3'<br>5'-GCCCTTGCTCACCATGCCTGAACCAGAGCCCTCGAgGAACAAAATAGGCTTTC-3'	N/A	Gibson assembly, This study
HA-CK1G3/pBSnHAcFL	HeLa total cDNA	5'-ACGCGTCGACAATGGAAAATAAAAAGAAAGACAAGG-3'<br>5'-GTACGGTACCGCGGCCGCTCATTTGTGGCGCTGTAT-3'	SalI/KpnI	This study
HA-CK1G3/pMXs-IRES-IN	HA- CK1G3/ pBSnHAcFL	N/A	EcoRI/NotI	This study
HA-CK1G3 ΔC38 /pMXs-IRES-IN	HA- CK1G3/ pBSnHAcFL	5'-GCAGAATTCACCATGTACCC-3'<br>5'-GTACGCGGCCGCTCAGGTGGGGTCATCTGTGTTTA-3'	EcoRI/NotI	This study
HA-CK1G3 ΔC20/ pMXs-IRES-IN	HA- CK1G3/ pBSnHAcFL	5'-GCAGAATTCACCATGTACCC-3'<br>5'-TTCGCGGCCGCTCAATCCATCACTTCTACTTCAGT-3'	EcoRI/NotI	This study
HA-CK1G3 K72R/ pMXs-IRES-IN	HA- CK1G3/ pMXs-IRES-IN	5'-TTTATACACAAATGAATATGTGGCAATTAGGTTGGAGCCCATGAAA-3'<br>5'-TTTCATGGGCTCCAACCTAATTGCCACATATTCATTTGTGTATAAA-3'	N/A	Site-directed mutagenesis, This study
HA-CK1G3 ΔC38 K72R/ pMXs-IRES-IN	HA- CK1G3 K72R/ pMXs-IRES-IN	5'-GCAGAATTCACCATGTACCC-3'<br>5'-GTACGCGGCCGCTCAGGTGGGGTCATCTGTGTTTA-3'	EcoRI/NotI	This study
HA-CK1G3 ΔC20 K72R/ pMXs-IRES-IN	HA-CKIγ3 K72R/ pMXs-IRES-IN	5'-GCAGAATTCACCATGTACCC-3'<br>5'-TTCGCGGCCGCTCAATCCATCACTTCTACTTCAGT-3'	EcoRI/NotI	This study
HA-CK1G3 3CA/ pMXs-IRES-IN	HA-CK1G3/ pMXs-IRES-IN	5'-CTACTGAAGTAGAAGTGATGGATGAAACCAAGGCCGCCGCTTTTTTCAAACGAA GGAAAAGGAAAACCATAC-3'	N/A	Site-directed mutagenesis, This

		5'-GTATGGTTTTCCTTTTCCTTCGTTTGAAAAAAGCGGCGGCCTTGGTTTCATCCATCACTTCTACTTCAGTAG-3'		study
HA-CK1G2/pMXs-IRES-IN	N/A	N/A	N/A	Tomishige et al., 2009
HA-CK1G2 ΔC20/pMXs-IRES-IN	HA-CK1G2/pMXs-IRES-IN	5'-TACAGAATTCGGCACGAGCAGCAGAATGTC-3'<br>5'-ATCCCTCGAGTCAATCGGCCACCTCCACCTCTG-3'	EcoRI/XhoI	This study
HA-CK1G1/pMXs-IRES-IN	HA-CK1G1/pCMV3 (Sino Biological)	5'-GCATGAATTCATGTATCCTTACGACGTGCC-3'<br>5'-GCATGCGGCCGCTTACTTGTGGCGCTGAGCAG-3'	EcoRI/NotI	This study
HA-CK1G1 ΔC20/pMXs-IRES-IN	HA-CK1G1/pCMV3 (Sino Biological)	5'-GCATGAATTCATGTATCCTTACGACGTGCC-3'<br>5'-TAGCGCGGCCGCTCACTCCACTACCTCCACCTCGG-3'	EcoRI/NotI	This study
